# Genomic and evolutionary aspects of phytoplasmas

**DOI:** 10.3389/fmicb.2013.00230

**Published:** 2013-08-14

**Authors:** Kenro Oshima, Kensaku Maejima, Shigetou Namba

**Affiliations:** Department of Agricultural and Environmental Biology, Graduate School of Agricultural and Life Sciences, The University of TokyoYayoi, Bunkyo-ku, Tokyo, Japan

**Keywords:** phytoplasma, genome, mycoplasma, reductive evolution, ATP synthase, secreted protein, virulence factor

## Abstract

Parasitic bacteria that infect eukaryotes, such as animals and plants, often have reduced genomes, having lost important metabolic genes as a result of their host-dependent life cycles. Genomic sequencing of these bacteria has revealed their survival strategies and adaptations to parasitism. Phytoplasmas (class Mollicutes, genus ‘*Candidatus* Phytoplasma’) are intracellular bacterial pathogens of plants and insects and cause devastating yield losses in diverse low- and high-value crops worldwide. The complete genomic sequences of four *Candidatus* Phytoplasma species have been reported. The genomes encode even fewer metabolic functions than other bacterial genomes do, which may be the result of reductive evolution as a consequence of their life as an intracellular parasite. This review summarizes current knowledge of the diversity and common features of phytoplasma genomes, including the factors responsible for pathogenicity.

## INTRODUCTION

**FIGURE 1:**
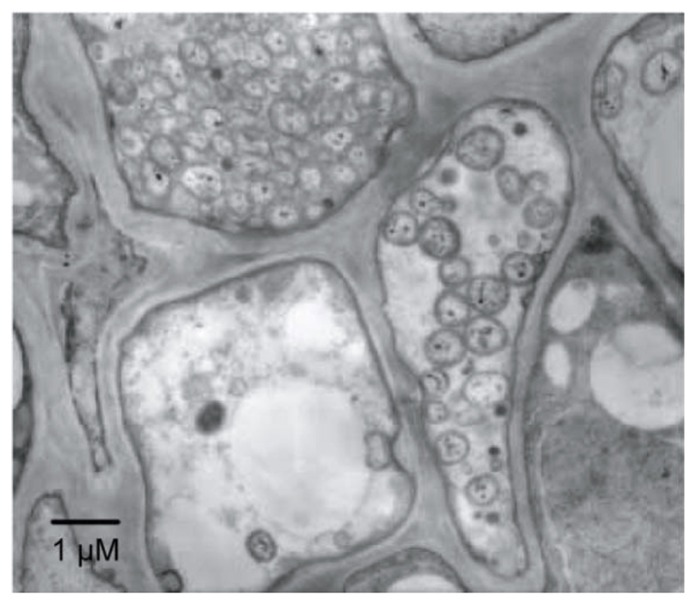
**Electron microphotograph of *Candidatus* Phytoplasma asteris infecting the plant phloem.** A section of phloem tissue from an infected plant vascular bundle is shown. Several of the plant cells contain phytoplasma cells.

**FIGURE 2:**
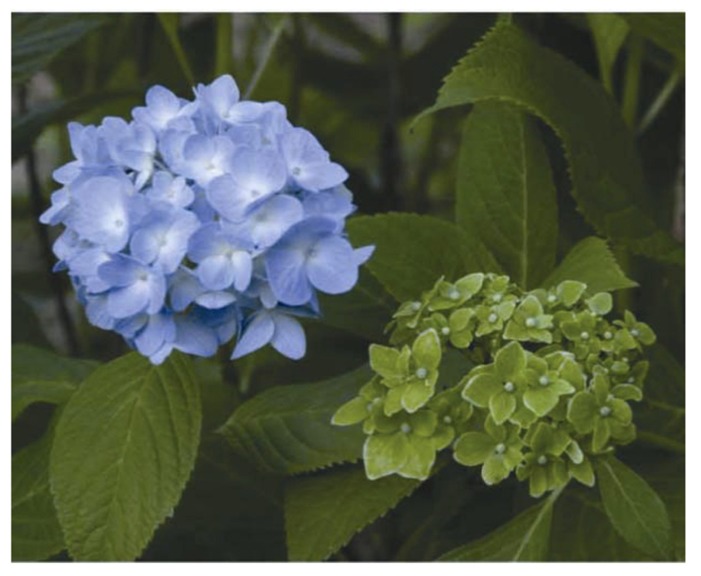
**Typical symptoms in a phytoplasma-infected hydrangea (right), called “phyllody”.** A healthy flower is shown on the left.

Phytoplasmas (genus ‘*Candidatus* Phytoplasma’) are plant pathogens of the bacterial class Mollicutes (Lee et al., 2000; Hogenhout et al., 2008). Phytoplasmas lack rigid cell walls, are surrounded by a single membrane, and are spherical or pleiomorphic with sizes similar to those of mycoplasmas (80–800 nm; **Figure 1**); therefore, phytoplasmas were called mycoplasma-like organisms upon their discovery in 1967 (Doi et al., 1967). Sequence analysis of 16S rRNA and other housekeeping genes suggest that phytoplasmas are members of the class Mollicutes but are more closely related to the *Acholeplasma* spp. than to the *Spiroplasma* spp. or animal mycoplasmas (Lim and Sears, 1992; Namba et al., 1993; Oshima and Nishida, 2007). Phytoplasmas are transmitted by insect vectors and infect over 700 plant species, including many economically important crops such as fruit trees and ornamental plants (Bertaccini, 2007). Infected plants show a wide range of symptoms, including stunting, yellowing, witches’ broom (proliferation of shoots), phyllody (formation of leaf-like tissues instead of flowers; **Figure 2**), virescence (greening of floral organs), proliferation (growth of shoots from floral organs), purple top (reddening of leaves and stems), and phloem necrosis (Hogenhout et al., 2008).

Phytoplasma infection is often fatal and causes devastating damage to agricultural production around the world. For example, phytoplasmal epidemics among coconut palms have destroyed the livelihoods of many people in Africa and the Caribbean, who depend on the trees for nourishment, building materials, and income (Strauss, 2009). In 2001, a phytoplasma outbreak in apple trees caused a loss of about €100 million in Italy (Strauss, 2009). In addition, phytoplasma-related diseases are expected to increase because global warming/climate change is advantageous to the cold-sensitive phytoplasma vectors (Hogenhout et al., 2008). Therefore, the development of phytoplasma pest control methods will become more important in the future.

Phytoplasmas parasitize the phloem tissues of infected plant hosts and are transmitted by insect vectors (mostly leafhoppers; Christensen et al., 2005; Hogenhout et al., 2008). After infecting an insect, the bacteria traverse the wall of the intestinal tract, multiply in the hemolymph, pass through the salivary glands, and multiply further. When the infected insect feeds on a new host plant, the phytoplasmas are introduced into the phloem tissue along with salivary fluids.

**FIGURE 3:**
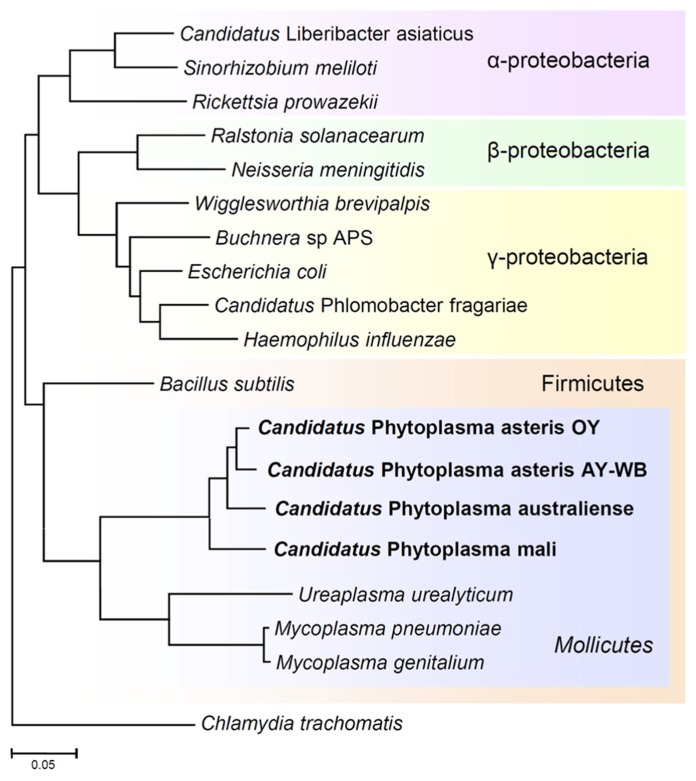
**Phylogenetic distance tree constructed by the neighbor-joining method, comparing the 16S rRNA gene sequences of phytoplasmas with those of other bacteria obtained from GenBank.** The sequence of *Chlamydia trachomatis* was used as an out-group to root the tree. Phytoplasmas are currently classified as Mollicutes, along with mycoplasmas.

Although the unique features of phytoplasmas have long made them a subject of interest, the difficulty of *in vitro* culture has hindered their molecular characterization. In the past decade, whole genome sequences have been completed for several phytoplasma strains (**Figure 3**; Oshima et al., 2004; Bai et al., 2006; Kube et al., 2008; Tran-Nguyen et al., 2008), enabling better understanding of the molecular mechanism underlying virulence and host interaction (Suzuki et al., 2006; Oshima et al., 2007, 2011; Hoshi et al., 2009). In this review, we summarize the history and recent progress in phytoplasma research from genomic and evolutionary perspectives.

## UNIQUE FEATURES OF PHYTOPLASMA GENOMES

**FIGURE 4:**
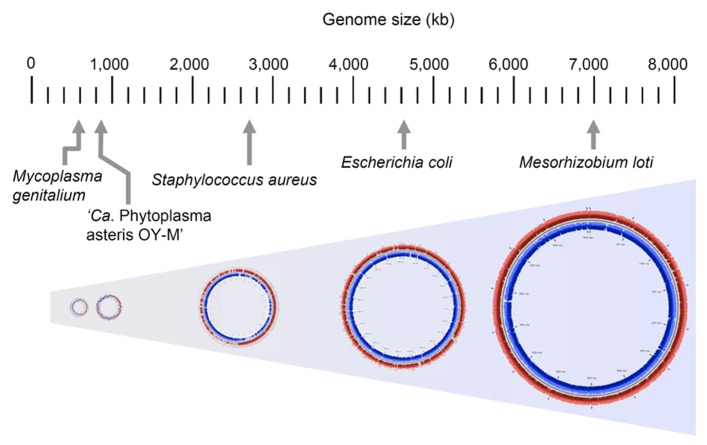
**Comparison of genome size in phytoplasma and other bacteria.** Genome sizes of *Mesorhizobium loti*, *Escherichia coli* K-12 MG1655, *Staphylococcus aureus* subsp. *aureus* N315, ‘*Ca*. P. asteris’ OY-M strain and *Mycoplasma genitalium* G37 are indicated.

To characterize the genomic features of phytoplasma, several genome projects were begun all over the world in the 1990s and some genomic fragments have been cloned (Miyata et al., 2002, 2003; Oshima et al., 2002). The complete genomic sequence of ‘*Ca*. P. asteris’ OY-M strain was first determined in 2004 (Oshima et al., 2004); since then, the complete genomic sequences of ‘*Ca*. P. asteris’ AY-WB strain, ‘*Ca*. P. australiense’ and ‘*Ca*. P. mali’ have been reported (Bai et al., 2006; Kube et al., 2008; Tran-Nguyen et al., 2008). In general, a phytoplasma genome consists of one chromosome and several small plasmids with a unique replication gene (Nishigawa et al., 2001; Oshima et al., 2001a; Firrao et al., 2007), although ‘*Ca*. P. mali’ harbors no plasmids (Kube et al., 2012). The phytoplasma chromosome size is 600–880 kb, which is quite small in comparison to those of other plant pathogens but similar to those of mycoplasmas (**Figure 4**). Phytoplasma genomes have a low G+C content (21–28%), similar to mycoplasmas (Glass et al., 2000) and endosymbiotic bacteria (Tamas et al., 2002; Wernegreen, 2002). Since the small genomes of parasitic and/or symbiotic bacteria are comprised mostly of functional genes, comparisons of the metabolic pathways in these organisms often reveal fundamental divergences in the microbial way of life and their evolutionary origins (Moran, 2002). In general, small-genome pathogenic bacteria have lost the genes for numerous biosynthetic pathways, most likely because many metabolites are available within the host cell environment; this reduces the selective constraints on genes for biosynthetic capabilities. In addition, selection favoring the loss of factors such as microbe/pathogen-associated molecular patterns (MAMPs or PAMPs) that may trigger host responses (Jones and Dangl, 2006) is another likely explanation for gene loss events, especially for phytoplasmas that must navigate between two diverse hosts (Hogenhout et al., 2008).

Although the phytoplasma genome contains genes for basic cellular functions such as DNA replication, transcription, translation, and protein translocation (Kakizawa et al., 2001; Jung et al., 2003), it has lost many metabolic genes. In general, mycoplasmas lack genes for the tricarboxylic acid cycle, sterol biosynthesis, fatty acid biosynthesis, *de novo* nucleotide synthesis, and biosynthesis of most amino acids; thus, they must depend entirely on their host to supply them with the products of these pathways (Razin et al., 1998). Similarly, no genes for these biosynthetic pathways have been identified in phytoplasmas. However, the phytoplasmas have lost more metabolic genes than the mycoplasmas (Oshima et al., 2004; Bai et al., 2006), including those of the pentose phosphate pathway. Instead, phytoplasmas harbor multiple copies of transporter-related genes that are not found in mycoplasmas (Oshima et al., 2004). These genomic features suggest phytoplasmas are highly dependent on metabolic compounds from their host.

## ABSENCE OF THE F_1_F_o_-TYPE ATP SYNTHASE

The most unique feature of the phytoplasma genome may be the absence of the gene encoding F_1_F_o_-type ATP synthase. In general, bacteria use F_1_F_o_-type ATP synthases to synthesize and hydrolyze ATP using ATP-proton motive force interconversion. Like other eubacteria, mycoplasmas also possess an F_1_F_o_-type ATP synthase (Razin et al., 1998); however, no genes for an F_1_F_o_-type ATP synthase have been identified in the four sequenced phytoplasmas (Oshima et al., 2004; Bai et al., 2006), suggesting these genes may have been lost. Since the genes encoding ATP synthase have been found in most fully sequenced bacteria, ‘*Ca*. P. asteris’ OY-M strain was the first example of a naturally occurring organism with no ATP synthase genes (Oshima et al., 2004).

Despite the absence of an ATP synthase gene, there is considerable membrane potential in phytoplasmas, as has been demonstrated with potentiometric dye (Christensen et al., 2004). Phytoplasmas have five genes encoding P-type ATPases, which are similar to animal Na^+^/K^+^ and H^+^/K^+^ pumps (Bai et al., 2006), implying that these ATPases may generate electrochemical gradients across the membrane (Christensen et al., 2005).

## GLYCOLYTIC PATHWAY

In light of a previous report showing that glycolytic turnover increases in *Bacillus subtilis* strains in which the *atp* operon has been deleted (Santana et al., 1994), ATP synthesis in phytoplasma is likely to be strongly dependent on the glycolytic pathway. Dependence on the glycolytic pathway is also supported by sequencing analysis of ca. 80-kb genomic DNA from ‘*Ca*. P. asteris’ OY-W strain, which causes severe symptoms (Oshima et al., 2001b). Interestingly, an approximately 30-kb region was found to be tandemly duplicated in the ‘*Ca*. P. asteris’ OY-W strain genome (Oshima et al., 2007). Two sets of five glycolytic enzymes were encoded in this genomic region, which is a unique gene structure not identified in any other bacterial genomes. The gene organization of glycolytic genes of ‘*Ca*. P. asteris’ AY-WB strain (Bai et al., 2006) is similar to that of ‘*Ca*. P. asteris’ OY-M strain rather than ‘*Ca*. P. asteris’ OY-W strain, suggesting the duplication of glycolytic genes was specific to ‘*Ca*. P. asteris’ OY-W strain.

Glycolysis seems to be an important metabolic pathway in phytoplasmas, although the genes for glycolysis are completely absent in ‘*Ca*. P. mali’ (Kube et al., 2008), which instead carries the gene for 2-dehydro-3-deoxyphosphogluconate aldolase (*eda*; Kube et al., 2012), unlike the other three sequenced phytoplasma strains. An alternative metabolic pathway has been hypothesized in ‘*Ca*. P. mali’, in which pyruvate is formed independently from glycolysis (Kube et al., 2012). Further studies are needed to elucidate the energy-yielding systems of phyto-plasmas.

## DIVERSITY AND COMMON FEATURES OF METABOLIC GENES

**FIGURE 5:**
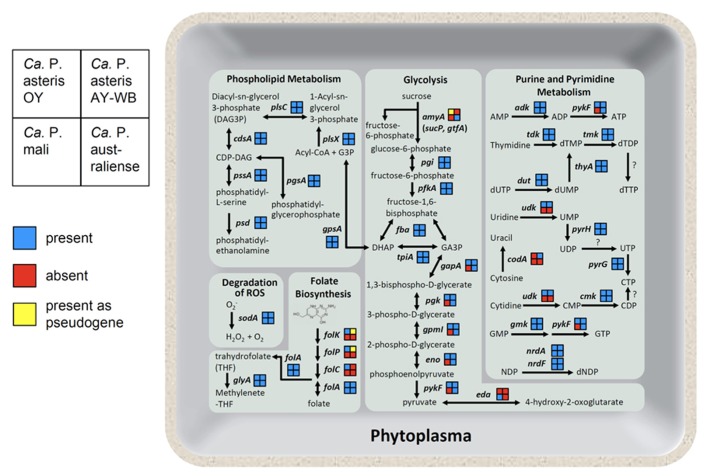
**Overview of the phytoplasma metabolic pathways.** As shown on the left, the gene content in each phytoplasma strain is indicated as presence (blue), absence (red), or presence as a pseudogene (yellow). Abbreviated gene names are as follows: *amyA*, sucrose phosphorylase; *pgi*, glucose-6-phosphate isomerase; *pfkA*, 6-phosphofructokinase; *fba*, fructose bisphosphate aldolase; *tpiA*, triosephosphate isomerase; *gapA*, glyceraldehyde-3-phosphate dehydrogenase; *pgk*, 3-phosphoglycerate kinase; *gpmI*, phosphoglyceromutase; eno, enolase; *pykF*, pyruvate kinase; *eda*, 2-dehydro-3-deoxyphosphogluconate aldolase; *gpsA*, glycerol 3-phosphate dehydrogenase; *plsX*, fatty acid/phospholipid biosynthesis enzyme; *plsC*, 1-acyl-sn-glycerol-3-phosphate acyltransferase; *cdsA*, CDP-diglyceride synthetase; *pssA*, phosphatidylserine synthase; *psd*, phosphatidylserine decarboxylase; *pgsA*, phosphatidylglycerophosphate synthase; *sodA*, superoxide dismutase; *folk*, 7, 8-dihydro-6-hydroxymethylpterin- pyrophosphokinase; *folP*, dihydropteroate synthase; *folC*, folylpolyglu-tamate synthase; *folA*, dihydrofolate reductase; *glyA*, glycine hydroxymethyltransferase; *adk*, adenylate kinase; *tdk*, thymidine kinase; *tmk*, thymidylate kinase; *thyA*, thymidylate synthase; *dut*, dUTPase; *udk*, uridine kinase; *pyrH*, uridylate kinase; *pyrG*, CTP synthase; *codA*, cytosine deaminase; *cmk*, cytidylate kinase; *gmk*, guanylate kinase; *nrdA*, ribonucleotide reductase alpha subunit; *nrdF*, ribonucleotide reductase beta subunit.

Phytoplasma genomes are highly diverse in genome structure and content (**Figure 5**; also reviewed in Kube et al., 2012). The uptake of carbohydrate from host cells is an important feature of phytoplasma. Sucrose seems to be an ideal carbon source for bacteria inside a plant, as the highest concentrations of sucrose (200–1,600 mM) are found in the sieve tubes. A gene for sucrose phosphorylase (*amyA*, synonym *sucP* or *gtfA*), which cleaves sucrose into glucose and fructose, was identified in the ‘*Ca.* P. australiense’ genome. The *amyA* gene was also identified in the ‘*Ca*. P. asteris’ OY-M strain genome; however, as it contains a mid-sequence frameshift mutation leading to an early termination codon, the expected ORF is incomplete and is divided into two smaller ORFs (Oshima et al., 2004), suggesting that an ancestral phytoplasma may have possessed a functional copy of this gene. In contrast, the ‘*Ca*. P. mali’ and ‘*Ca*. P. asteris’ AY-WB strain genomes do not harbor *amyA* (**Figure 5**). These findings suggest utilization of carbon sources may differ between phytoplasma species.

Although metabolic genes are reduced, phytoplasma genomes encode several genes necessary for folate biosynthesis. Four genes involved in folate biosynthesis (*folk*, *folP*, *folC*, and *folA*) are encoded in the ‘*Ca*. P. asteris’ OY-M strain genome. In contrast, only *folA* is encoded in the ‘*Ca*. P. mali’ and ‘*Ca*. P. australiense’ genomes. In the ‘*Ca*. P. asteris’ AY-WB strain genome, the *folA* gene is complete, but *folk* and *folP* are pseudogenes (**Figure 5**). This diversity in gene content suggests that at least four genes for folate biosynthesis may have been encoded in an ancestral phytoplasma genome but were gradually eliminated in the course of its reductive evolution.

Although no genes for *de novo* synthesis of nucleotides are encoded in the phytoplasma genomes, they do contain genes for salvage pathways of purine and pyrimidine metabolism, more similar to *Rickettsia* than mycoplasmas (Oshima et al., 2004). Gene content associated with purine and pyrimidine metabolism also differs between phytoplasma species. The gene for cytidine/uridine kinase is encoded in ‘*Ca*. P. asteris’ OY-M strain and ‘*Ca*. P. asteris’ AY-WB strain but not in ‘*Ca*. P. mali’ or ‘*Ca*. P. australiense.’ Interestingly, only ‘*Ca*. P. asteris’ AY-WB strain possesses the gene for cytosine deaminase (*codA*; **Figure 5**).

In contrast to the diversified energy metabolic genes, all four phytoplasma genomes contain the *sodA* gene (**Figure 5**), which encodes Mn-SOD, a protein that can inactivate reactive oxygen species (ROS; Miura et al., 2012). Plants deploy a broad range of defenses during infection by various pathogens. The oxidative burst, which produces ROS, is one of the earliest events in the plant defense response. Since the genomes of mycoplasmas do not contain this gene, the presence of *sodA* may help phytoplasmas defend themselves against the unique threat of ROS released by the plant cell.

## POTENTIAL MOBILE UNITS IN PHYTOPLASMA GENOMES

Intriguingly, phytoplasma genomes contain clusters of repeated gene sequences, named potential mobile units (PMUs; Bai et al., 2006) or sequence-variable mosaics (SVMs; Jomantiene and Davis, 2006; Jomantiene et al., 2007; Wei et al., 2008). PMUs and SVMs have similar compositions and contain similar genes; henceforth, these gene clusters have been referred to as PMUs in this review. In the ‘*Ca*. P. asteris’ AY-WB strain genome, PMUs are ~20 kb in size and consist of genes with similarities to *sigF*, *hflB*, *dnaG*, *dnaB*, *tmk*, *ssb*, *himA*, and the IS3 family insertion sequence tra5, organized in a conserved order (Bai et al., 2006). These genes are also found in multiple copies, singly or in clusters, in other phytoplasma genomes (Oshima et al., 2004; Lee et al., 2005; Jomantiene and Davis, 2006; Jomantiene et al., 2007; Arashida et al., 2008). The repeated presence of PMUs, their gene contents, including genes for recombination (*tra5*, *ssb*, *himA*) and replication (*dnaG*, *dnaB*), and their conserved gene order suggests the PMUs are replicative composite transposons (Bai et al., 2006; Arashida et al., 2008). The PMU exists as linear chromosomal and circular extrachromosomal elements in ‘*Ca*. P. asteris’ AY-WB strain (Toruno et al., 2010), suggesting that it has the ability to transpose within the genome. The presence of multiple PMUs or apparently degenerated PMU-like sequences, such as SVMs, and the dramatic loss of basic metabolic pathways in phytoplasma genomes (Oshima et al., 2004; Bai et al., 2006) suggest PMUs are likely to be important for phytoplasma fitness.

## PROTEIN SECRETION

Since phytoplasmas have no cell wall and reside inside of host cells, their membrane proteins and secreted proteins function in the cytoplasm of the host plant or insect cell, and are predicted to have important roles in host–parasite interactions and/or virulence. Thus, the identification of both a secretion system and secreted proteins in phytoplasma genomes is important for understanding the biology of phytoplasmas. Phytoplasmas possess two secretion systems, the YidC system for the integration of membrane proteins, and the Sec system for the integration and secretion of proteins into the host cell cytoplasm.

The Sec protein translocation system is essential for viability in many bacteria (Economou, 1999; Tjalsma et al., 2000). The *Escherichia coli* Sec system, which is composed of at least 11 proteins and 1 RNA species, is the well-characterized Sec system (Economou, 1999). Among these proteins, SecA, SecY, and SecE are essential for protein translocation and cell viability in *E. coli* (Economou, 1999), and protein translocation activity can be reconstituted *in vitro* with only these three proteins (Akimaru et al., 1991). Genes encoding SecA, SecY, and SecE have been identified in the ‘*Ca*. P. asteris’ OY-M strain genome, the (Kakizawa et al., 2001, 2004), and SecA expression has been confirmed in phytoplasma-infected plants (Kakizawa et al., 2001; Wei et al., 2004). These genes have also been identified in three other phytoplasma genomes (Bai et al., 2006; Kube et al., 2008; Tran-Nguyen et al., 2008), and *secY* genes have been cloned from many phytoplasma strains (Lee et al., 2006). These results strongly suggest that a functional Sec system is common among phytoplasmas.

Antigenic membrane protein (Amp), a major surface membrane protein of phytoplasmas (Barbara et al., 2002), has been reported to be a substrate of the Sec system. Amp has a Sec signal sequence at its N-terminus, which is cleaved in ‘*Ca*. P. asteris’ OY-M strain (Kakizawa et al., 2004), suggesting that the phytoplasma Sec system utilizes recognition and cleavage of a signal sequence, as in other bacterial Sec systems. This finding also suggests that signal sequence prediction programs, such as SignalP (Nielsen et al., 1997) or PSORT (Nakai and Kanehisa, 1991), may be applicable to phytoplasmal proteins and could be used to identify secretory proteins (Kakizawa et al., 2004). Several studies were performed to identify phytoplasma secretory proteins in their genome sequences, as given below.

YidC mediates integration of newly synthesized membrane proteins (Dalbey and Kuhn, 2000). Initially, YidC was found to co-purify with components of the Sec system (Scotti et al., 2000), and it was thought that YidC functions with the Sec system to insert transmembrane proteins into the lipid bilayer (Urbanus et al., 2001). However, YidC is sufficient to promote insertion of membrane proteins *in vitro*, suggesting its function is independent of the Sec system (Serek et al., 2004). YidC is encoded in all four completed phytoplasma genomes (Oshima et al., 2004; Bai et al., 2006; Kube et al., 2008; Tran-Nguyen et al., 2008); thus, phytoplasmas should have a YidC integration system. YidC is an essential protein in *E. coli* (Samuelson et al., 2000) and may also play an important role in phytoplasmas.

## PHYTOPLASMA VIRULENCE FACTORS

Many gram-negative pathogens of plants and animals possess Type III secretion systems (T3SSs) that can inject bacterial virulence “effector” proteins into host cells (Cornelis and Van Gijsegem, 2000). They are important for the pathogenicity of *Pseudomonas*, *Xanthomonas*, *Ralstonia*, *Erwinia*, and *Pantoea*. T3SSs and flagella are evolutionarily related and share a remarkably similar basal structure. T3SSs and flagella are restricted to gram-negative bacteria, and the gram-positive phytoplasmas therefore possess no T3SSs.

Since membrane and secreted proteins are potential virulence factors, the phytoplasma genomes have been mined for the presence of these proteins. Among the secreted proteins of ‘*Ca*. P. asteris’ OY-M strain, TENGU has been reported to induce symptoms similar to phytoplasma infection, including witches’ broom (development of numerous shoot branches) and dwarfism (Hoshi et al., 2009; Sugawara et al., 2013). TENGU encodes a very small protein (4.5 kDa). The mature protein, after cleavage of the N-terminal signal peptide, is only 38 amino acids in length. Microarray analyses revealed that the expression of many auxin-related genes was significantly downregulated in tengu-transgenic plants, suggesting that TENGU suppresses auxin signaling or biosynthesis pathways (Hoshi et al., 2009; Denancé et al., 2013). It has been also reported that phytoplasma-infected periwinkles can show remission of disease symptoms when cultured in medium containing a high-concentration of auxin (Pertot et al., 1998; Curkovic Perica et al., 2007). Thus, auxin may have a great impact on the plant–phytoplasma interaction.

In the ‘*Ca*. P. asteris’ AY-WB strain genome, more than 56 genes encode predicted secreted proteins (Bai et al., 2009). Among them, SAP11 contains eukaryotic nuclear localization signals and localizes in plant cell nuclei (Bai et al., 2009). SAP11-expressing plants exhibit crinkled leaves and produce many stems (Sugio et al., 2011a). Moreover, the fecundity of insect vectors was increased on SAP11-expressing versus normal plants (Sugio et al., 2011a). Thus, phytoplasma-secreted proteins may manipulate the host and mediate virulence, similar to the findings seen for other pathogens (Higgins, 2001; Boutareaud et al., 2004). In addition to SAP11, SAP54 of ‘*Ca*. P. asteris’ AY-WB strain was reported to cause morphological changes in *Arabidopsis thaliana* flower organ development, similar to the symptoms observed in phytoplasma-infected plants (MacLean et al., 2011). Although the molecular mechanisms remain unknown, it is assumed that proteins secreted by phytoplasmas may interfere with the function of genes involved in flower development (Sugio et al., 2011b; Sugio and Hogenhout, 2012).

Researchers believed phytoplasma disease symptoms are caused by the side effects of infection (indirect effects), such as the consumption of metabolites of infected plants. This idea was partially supported by the absence of many metabolic pathways and metabolite synthesis, as well as the retention of transporter genes to absorb metabolites and nutrients from the host cytoplasm (Oshima et al., 2004, 2007). However, the identification of TENGU, SAP11, and SAP54 clearly changed this idea: phytoplasma could aggressively induce symptoms by secretion of effector proteins and modification of plant-gene activity (Hoshi et al., 2009; Sugio et al., 2011a; Himeno et al., 2011). Therefore, additional searches for effector proteins in phytoplasma genomes would be useful.

## TRANSCRIPTIONAL CHANGES DURING HOST SWITCHING BETWEEN PLANT AND INSECT

Since phytoplasmas are intracellular parasites of both plants and insects (Christensen et al., 2005), their ability to adapt to two diverse environments is of considerable interest. Microarray analysis of ‘*Ca*. P. asteris’ OY-M strain revealed that expression of approximately 33% of the genes changes during host switching between plant and insect, suggesting phytoplasma dramatically alters gene expression in response to its host (Oshima et al., 2011) and may use transporters, secreted proteins, and metabolic enzymes in a host-specific manner.

The genes encoded in the PMU of ‘*Ca*. P. asteris’ AY-WB strain are more highly expressed in insects than in plants (Toruno et al., 2010), most likely due to increased production of the extrachromosomal circular type of PMU during insect infection (Toruno et al., 2010). Differential gene expression between plant and insect hosts has been also reported in ‘*Ca*. P. asteris’ OY-M strain, in which TENGU is more highly expressed in plant hosts than in insect hosts (Hoshi et al., 2009). As phytoplasmas reside within the host cell, secreted proteins are thought to play crucial roles in the interplay between pathogen and host cell. Therefore, the expression of virulence factors might be strictly regulated.

## FUTURE STUDY

Analysis of the phytoplasma genome revealed not only the reductive evolution as a consequence of its life as an intracellular parasite but also the virulence factors that induce symptoms unique to phytoplasma diseases. Further analysis of phytoplasma genomes will improve our understanding of these economically important and biologically attractive microorganisms.

Phytoplasmas cause various disease symptoms including witches’ broom, dwarfism, proliferation, phyllody, virescence, flower sterility, bolting, purple tops, generalized yellowing, and phloem necrosis (Bertaccini, 2007; Hogenhout et al., 2008). Three virulence factors have been identified, and the glycolytic pathway has been associated with some symptoms; however, not all phytoplasma disease symptoms could be explained by these virulence factors and pathways. The most probable candidates would be secreted proteins, so further functional analyses of secreted proteins of phytoplasma genomes are important, and further identification of virulence factors will help elucidate the pathogenic mechanisms and biology of phyto-plasmas.

## Conflict of Interest Statement

The authors declare that the research was conducted in the absence of any commercial or financial relationships that could be construed as a potential conflict of interest.
